# A large, general and modular DARPin–apoferritin scaffold enables the visualization of small proteins by cryo-EM

**DOI:** 10.1107/S2052252525003021

**Published:** 2025-04-25

**Authors:** Xin Lu, Ming Yan, Yang Cai, Xi Song, Huan Chen, Mengtan Du, Zhenyi Wang, Jia’an Li, Liwen Niu, Fuxing Zeng, Quan Hao, Hongmin Zhang

**Affiliations:** a Institute of High Energy Physics Chinese Academy of Sciences Beijing100000 People’s Republic of China; b Spallation Neutron Source Science Center Chinese Academy of Sciences Dongguan523000 People’s Republic of China; c Department of Biology, Southern University of Science and Technology, Shenzhen518055, People’s Republic of China; d School of Biomedical Sciences The University of Hong Kong Hong Kong People’s Republic of China; e The Chinese University of Hong Kong (Shenzhen) Shenzhen People’s Republic of China; f Hefei National Laboratory for Physical Sciences at the Microscale and School of Life Sciences University of Science and Technology of China Hefei230026 People’s Republic of China; g Key Laboratory of Structural Biology Chinese Academy of Sciences Hefei230026 People’s Republic of China; h Bio-Tech Center, Shenzhen Medical Academy of Research and Translation (SMART), Shenzhen518107, People’s Republic of China; Boston University School of Medicine, USA

**Keywords:** cryo-EM, small proteins, scaffolds, DARPin–apoferritin

## Abstract

Single-particle cryo-electron microscopy (cryo-EM) has emerged as an indispensable technique in structural biology that is pivotal for deciphering protein architectures. However, the medium-sized proteins (30–40 kDa) that are prevalent in both eukaryotic and prokaryotic organisms often elude the resolving capabilities of contemporary cryo-EM methods. To address this challenge, we engineered a scaffold strategy that securely anchors proteins of interest to a robust, symmetric base via a selective adapter. Our most efficacious constructs, namely models 4 and 6c, feature a designed ankyrin-repeat protein (DARPin) rigidly linked to an octahedral human apoferritin via a helical linker. By utilizing these large, highly symmetric scaffolds (∼1 MDa), we achieved near-atomic-resolution cryo-EM structures of green fluorescent protein (GFP) and maltose-binding protein (MBP), revealing nearly all side-chain densities of GFP and the distinct structural features of MBP. The modular design of our scaffold allows the adaptation of new DARPins through minor amino-acid-sequence modifications, enabling the binding and visualization of a diverse array of proteins. The high symmetry and near-spherical shape of the scaffold not only mitigates the prevalent challenge of preferred particle orientation in cryo-EM but also significantly reduces the demands of image collection and data processing. This approach presents a versatile solution, breaking through the size constraints that have traditionally limited single-particle cryo-EM.

## Abstract 2

This study introduces a modular scaffold strategy utilizing designed ankyrin-repeat proteins (DARPins) and a symmetric apoferritin base to overcome size limitations in single-particle cryo-EM, enabling near-atomic-resolution structural determination of medium-sized proteins like GFP and MBP. The high-symmetry, near-spherical scaffold not only resolves the common preferred-orientation challenges in single-particle cryo-EM but also reduces data-processing demands, offering a versatile platform for structural analysis of diverse proteins.

## 1. Introduction

Structural biology has made significant strides in unraveling the complexities of biological mechanisms and informing drug design, largely through the elucidation of biomolecular structures (Maveyraud & Mourey, 2020 ▸). However, the three cornerstone techniques of the field, X-ray crystallography, nuclear magnetic resonance (NMR) and cryo-electron microscopy (cryo-EM), each have their own set of limitations (Shi, 2014 ▸). X-ray crystallography, a workhorse for structural determination, often stumbles at the hurdle of crystallization, particularly for the elusive membrane proteins (Jones, 2014 ▸). NMR, a dynamic tool for probing protein motions, finds its scope confined to smaller proteins, typically under 40 kDa (Sekhar & Kay, 2019 ▸). Cryo-EM, an emerging powerhouse, has been instrumental in resolving near-atomic structures of complex macromolecular assemblies such as the ribosome (Jomaa *et al.*, 2016 ▸), spliceosome (Zhang *et al.*, 2018 ▸) and glycine receptor (Du *et al.*, 2015 ▸). Yet, despite its theoretical scope for molecules of up to 38 kDa (Henderson, 1995 ▸), cryo-EM is predominantly applied to proteins exceeding 150 kDa (Thompson *et al.*, 2016 ▸). This discrepancy is stark when considering the median molecular weights of eukaryotic and prokaryotic proteins, which are around 40 and 30 kDa, respectively (Brocchieri & Karlin, 2005 ▸). Thus, the quest to extend the reach of cryo-EM to smaller proteins is not just a scientific pursuit but is a necessity to fill a glaring void in structural biology.

The drive to visualize smaller proteins using cryo-EM has led to notable research milestones. Advances in sample preparation have pushed the boundaries, as demonstrated by the structures of the 64 kDa human hemoglobin protein, 67 kDa α-fetoprotein and 52 kDa streptavidin at 3.5 Å, 2.6 Å and 2.2 Å resolution, respectively. These achievements were made possible by the use of ultraflat graphene (UFG), which enhances the image quality of vitrified specimens (Zheng *et al.*, 2023 ▸). The innovative use of phase-plate technology has also yielded an ∼3.2 Å resolution structure of streptavidin (52 kDa; Fan *et al.*, 2019 ▸). Moreover, the engineering of large scaffold proteins to bind and stabilize small targets has opened new vistas for cryo-EM visualization (Yeates *et al.*, 2020 ▸). The successful implementation of this strategy hinges on two critical factors: rigidity to ensure high-resolution imaging and modularity to accommodate various target proteins without extensive reconstruction.

Recent efforts have concentrated on the development of scaffold systems, where a large core protein is tethered to an adaptor protein such as a nanobody or a designed ankyrin-repeat protein (DARPin). These adaptors provide a versatile platform for engaging a diverse array of small proteins (Wu & Rapoport, 2021 ▸; Castells-Graells *et al.*, 2023 ▸). In 2021, the advent of three nanobody-binding scaffolds marked a significant leap enabling cryo-EM structures of small proteins to be obtained in a resolution range of approximately 3.5–5 Å (Wu & Rapoport, 2021 ▸; Bloch *et al.*, 2021 ▸; Uchański *et al.*, 2021 ▸). However, the relatively modest size of these scaffolds (∼100 kDa) has somewhat restricted their broader application. More recently, DARPin-binding scaffolds have achieved near-atomic resolution for a small 26 kDa protein, green fluorescent protein (GFP), using cryo-EM (Castells-Graells *et al.*, 2023 ▸; Liu *et al.*, 2018 ▸, 2019 ▸; Vulovic *et al.*, 2021 ▸). However, the median sizes (250–600 kDa) and low symmetries [dihedral (Vulovic *et al.*, 2021 ▸) or tetrahedral (Liu *et al.*, 2018 ▸, 2019 ▸; Castells-Graells *et al.*, 2023 ▸)] of these scaffolds have posed limitations in their universal application in cryo-EM sample preparation and data processing. These strides underscore the need for further refinements to establish a robust and modular system for high-resolution imaging of small proteins.

In this study, we present a novel DARPin–apoferritin scaffold, with a larger size of around 1 MDa and higher octahedral symmetry, meticulously engineered for the binding and imaging of small target proteins via cryo-EM. Our innovative design has yielded resolutions of 3.47 Å for GFP and 4 Å for maltose-binding protein (MBP). This scaffold stands as a significant leap forward in the cryo-EM visualization of small proteins, promising to propel the field of structural biology into new realms of discovery.

## 2. Materials and methods

### 2.1. Cloning, protein expression and purification

To identify an appropriate scaffold, we selected the heavy chain of human apoferritin (PDB entry 3ajo; Masuda *et al.*, 2010 ▸) and DARPins targeting GFP (PDB entry 5ma8; Hansen *et al.*, 2017 ▸) and MBP (PDB entry 1svx; Binz *et al.*, 2004 ▸), along with an α-helical element (PDB entries 2o6n or 2zta; Sales *et al.*, 2007 ▸; O’Shea *et al.*, 1991 ▸), for analysis. DARPin–apoferritin scaffold constructs were engineered by fusing the DARPin to the N-terminus of apoferritin and incorporating the α-helical element at both the N-terminus of the DARPin and the C-terminus of apoferritin, thereby enhancing scaffold stability. Most constructs were synthesized by BGI Genomics and cloned into the pET-28a vector. Site-directed mutagenesis was performed using the QuickChange method to introduce mutations into these constructs. The DARPin–apoferritin scaffold, GFP (PDB entry 6nhv; Liu *et al.*, 2019 ▸) and MBP (PDB entry 1svx; Binz *et al.*, 2004 ▸) were individually expressed heterologously in SHuffle T7 *Escherichia coli* strain (Shanghai Weidi Biotechnology) and induced with 0.5 m*M* isopropyl β-d-1-thiogalactopyranoside at a temperature of 16°C during overnight incubation. Following cell lysis using a French press, the supernatant was purified using a HisTrap HP column (Cytiva), eluted with an imidazole gradient and the proteins were buffer-exchanged into binding buffer (20 m*M* Tris–HCl, 50 m*M* NaCl pH 8.0). The proteins were mixed in a 1:3 (scaffold:target) molecular ratio and incubated at 4°C overnight before undergoing size-exclusion chromatography using a Superose 6 Increase 10/300 GL column (Cytiva). The complex peak fractions were collected, concentrated to 1 mg ml^−1^ and characterized by SDS–PAGE, static light scattering and negative-stain electron microscopy (EM).

### 2.2. Static light scattering

Static light-scattering (SLS) measurements were performed using a miniDAWN TREOS instrument (Wyatt Technology) coupled to an ÄKTApure chromatography system (Cytiva) to determine the molecular weight of the protein complex. Protein samples, prepared at concentrations ranging from 3 to 5 mg ml^−1^, were loaded onto a pre-equilibrated Superose 6 Increase 10/300 GL column (Cytiva) using a Tris-based buffer (20 m*M* Tris–HCl, 50 m*M* NaCl pH 8.0). These procedures adhered to the established protocol for analytical size-exclusion chromatography (SEC; Cytiva).

### 2.3. Negative-stain EM

For the negative-stain electron microscopy (EM) analysis, 300 mesh Formvar–carbon-coated copper grids (Beijing Zhongjingkeyi Technology) were glow-discharged for 20–30 s. A 5 µl aliquot of the sample (0.1 mg ml^−1^) was deposited onto the prepared grids and allowed to adhere for 30 s. The grids were then stained with 5 µl 2% uranyl acetate for 30 s and air-dried at room temperature for 10 min. Sample visualization was conducted using a Talos L120C transmission electron microscope (TEM) from Thermo Fisher Scientific.

### 2.4. Cryo-EM specimen preparation and data collection

The DARPin–apoferritin scaffold protein was combined with excess GFP or MBP protein in a solution consisting of 50 m*M* Tris pH 8.0, 150 m*M* NaCl and incubated overnight. The protein complexes were subsequently isolated by gel filtration using a Superose 6 Increase 10/300 GL column (Cytiva), effectively separating and removing unbound GFP. The samples were concentrated to a concentration of 2 mg ml^−1^ and precipitates were eliminated by centrifugation. For cryo-EM, 3.5 µl of each sample was applied onto a glow-discharged M026-Au300-R20/20 copper grid (CryoMatrix) and blotted for 3 s at 8°C with 100% humidity. Subsequently, samples were plunge-frozen using a Vitrobot Mark IV (Thermo Fisher Scientific). Imaging was conducted on a Titan Krios G3 microscope (Thermo Fisher Scientific) equipped with a K2 Summit detector (Gatan). The Gatan imaging filter was adjusted to a slit width of 40 eV. The microscope was set to a nominal magnification of 130 000×, resulting in a calibrated pixel size of 1.076 Å per pixel. Data acquisition was automated using the *SerialEM* software, with defocus values set between −0.8 and −2.0 µm, and each exposure was limited to a cumulative dose of 50 e^−^ Å^−2^ distributed over 39 frames.

### 2.5. Image processing and 3D reconstruction

A total of 2922 movies were acquired in super-resolution mode for the GFP sample. Motion correction was performed using *MotionCor*2 (Zheng *et al.*, 2017 ▸) within *RELION* 3.1.2 (Scheres, 2012 ▸). CTF estimation was conducted using *CTFFIND*4 (Rohou & Grigorieff, 2015 ▸), resulting in 2878 micrographs being manually selected based on their CTF metrics. These micrographs were used for the automatic selection of particles using the *cryoSPARC* blob picker, yielding 750 123 particles. After 35 iterations of 2D classification in *cryoSPARC* 3.3.2 (Punjani *et al.*, 2017 ▸), 60 105 particles were selected for further 3D classification and refinement, leading to a 2.97 Å resolution map from 17 397 particles. This map revealed high-resolution details of the backbone, although the GFP structure was poorly reconstructed. To enhance the local resolution, the final particle stack from *cryoSPARC* 3.3.2 was then expanded with *O* symmetry using the *relion_particle_symmetry_expand* module within *RELION* 3.1.2. Expanded particles were applied to multiple 3D classifications with *C*1 symmetry. Subsequently, 113 239 particles exhibiting improved local resolution around the GFP region were chosen and were subtracted using a mask containing only one GFP molecule bound to the ‘dimeric’ DARPin. The subtracted particles were applied to multibody refinement (Nakane *et al.*, 2018 ▸), where the ‘dimeric’ DARPin was designated as the large body and the density representing the GFP molecule was designated as the small body. The data-processing procedure is detailed in Supplementary Table S1 and Fig. S1. The local resolution of the final map was determined using the *ResMap* program (Kucukelbir *et al.*, 2014 ▸).

In parallel, 2347 movies of the MBP sample were processed under similar conditions, resulting in the selection of 2047 images for particle picking. This process extracted 437 151 particles, which, after 23 rounds of 2D classification, were reduced to 91 926 for 3D analysis, culminating in a 2.69 Å resolution map that was subsequently sharpened in *cryo­SPARC* 3.3.2.

### 2.6. Model building and validation

The structural model of the DARPin–apoferritin backbone was built based on the known structures of human ferritin H chain (PDB entry 3ajo) and DARPins (anti-GFP, PDB entry 5ma8; anti-MBP, PDB entry 1svx). The ferritin and DARPin models were positioned within the density maps using the *Dock in Map* module of *Phenix* version 1.16 (Liebschner *et al.*, 2019 ▸) and then fine-tuned manually in *Coot* 0.9 (Emsley *et al.*, 2010 ▸). Subsequently, the GFP and MBP models were docked into the density map. The models were further refined and then manually adjusted in *Coot* 0.9.

## 3. Results

### 3.1. Screening and design of the DARPin–apoferritin scaffold

A previous report introduced a tetrahedral symmetric protein cage with an α-helical linker connecting the DARPin to the engineered scaffold. However, the initial design faced limitations due to the inherent flexibility of the α-helical linker, which impeded high-resolution imaging of the DARPin and its associated small protein cargoes (Liu *et al.*, 2018 ▸, 2019 ▸). To address these challenges, this tetrahedral cage scaffold was further refined with a modified α-helical linker that enhanced the orientation of the protruding arms, thereby facilitating closer contact. This innovative scaffold design allows three DARPins to converge at each vertex of the tetrahedron, thereby stabilizing both the DARPin and its associated small protein (Castells-Graells *et al.*, 2023 ▸).

**Figure 1:**
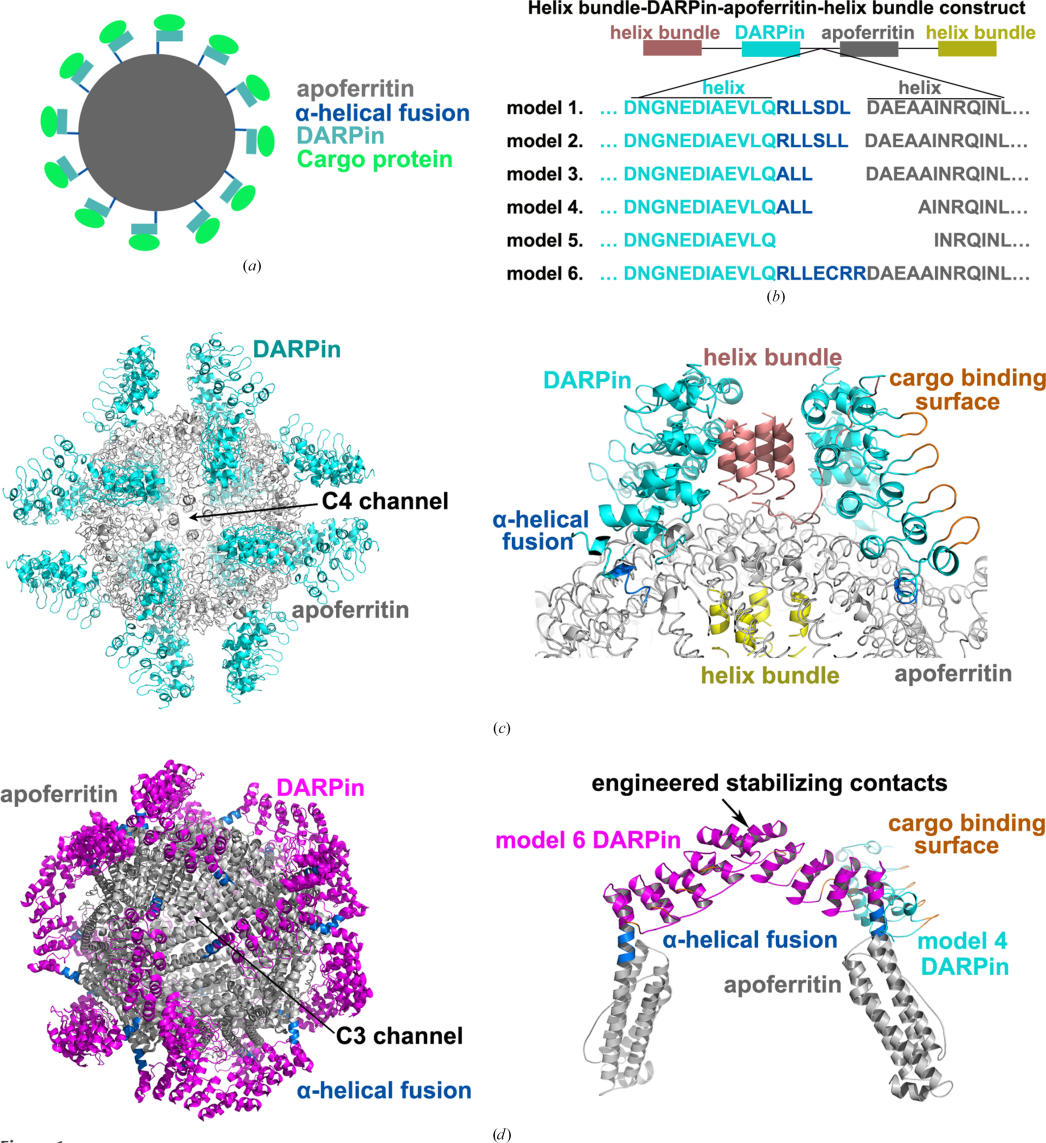
Design of the helix bundle–DARPin–apoferritin–helix bundle scaffold. (*a*) The scaffold is depicted as a cartoon, with apoferritin forming the inner cage and the DARPin serving as a platform for binding small cargo proteins on the outer portion. (*b*) The screening process for DARPin–apoferritin fusion constructs (model 1–6) is illustrated, with each construct and its junction sequences presented in distinct colors. (*c*, *d*) A detailed view of (*c*) model 4 and (*d*) model 6, specifically designed and characterized in this study, are shown. The apoferritin core of the scaffold, colored in gray, is prominently displayed as 24 subunits arranged in octahedral 432 symmetry. The N-terminal α-helix of apoferritin is connected to the C-terminal α-helix of the DARPin subunit (cyan in model 4 and magenta in model 6) through a continuous α-helix depicted in blue. The variable cargo-binding surface of the DARPin is highlighted in orange. In model 4, four neighboring DARPin molecules are oriented towards the C4 channel of the apoferritin cage. The helix-bundle part is designed to stabilize the scaffold at the N-terminus of the DARPin (deep salmon) and the C-terminus of apoferritin (yellow), as shown in (*c*) (right). Additionally, several mutations are introduced at the interface between the DARPin and apoferritin to enhance rigidity. In model 6, the N-termini of two DARPin subunits tightly bind around the C3 channel of apoferritin due to the extended and rotated α-helical fusion compared with that in model 4 (*d*).

In this study, we present a novel DARPin–apoferritin scaffold where the DARPin (anti-GFP; PDB entry 5ma8) is genetically fused at the N-terminus to the 24-subunit, octahedrally arranged apoferritin (PDB entry 3ajo) through a continuous α-helix. This scaffold is designed to specifically allow the small target protein GFP to bind to the DARPin as depicted in Fig. 1 ▸(*a*) and Section S1.

To develop a stable DARPin–apoferritin scaffold, we explored three regions of the DARPin–apoferritin constructs. Firstly, to identify an appropriate linker between the DARPin and apoferritin, we integrated a variable-length connection sequence to form a chimeric rigid α-helix between the DARPin and apoferritin across models 1–6 [Fig. 1 ▸(*b*)]. To minimize potential flexibility in the scaffolds, we performed truncations at the C-terminus of the DARPin (residues 159–162) and the N-terminus of apoferritin (residues 1–14). Secondly, to improve overall stability, we screened and introduced short α-helices at the N-terminus of the DARPin (PDB entry 2o6n, residues 18–34) and the C-terminus of apoferritin (PDB entry 2o6n, residues 2–17), creating a helix-bundle motif along with three other short α-helices [Figs. 1 ▸(*b*) and 1 ▸(*c*), Supplementary Fig. S2 and Section S1]. An additional linker was engineered to promote four-helix-bundle formation. Finally, mutations were strategically introduced at the DARPin–DARPin and DARPin–apoferritin interfaces to strengthen their interaction between the back face of the DARPin and the outer surface of apoferritin, while reducing steric hindrance at the DARPin–DARPin and DARPin–apoferritin interfaces (Sections S1 and S2).

All candidate models (1–6), except model 5, were successfully expressed in the *E. coli* system and assembled into a 24-subunit structure in 100 m*M* NaCl buffer, as confirmed by SDS–PAGE and static light-scattering analysis. Models 3, 4 and 6 exhibited a ring structure with a diameter of 120 Å, featuring additional density at the outer shell edge, matching the expected dimensions from negative-staining EM analysis. Cryo-EM analysis revealed that the apoferritin core in these three models achieved a local resolution between 2 and 2.5 Å. Notably, the DARPin component of model 4 showed a resolution superior to 4 Å, with the GFP target resolution ranging from 3.5 to 4 Å, indicating the need for further refinement (Supplementary Table S2).

### 3.2. Optimization and analysis of the DARPin–apoferritin scaffold

In our comparative analysis of the structures of models 4 and 6, we noticed an unexpected outward rotation in the linker that connects apoferritin to the DARPin, resulting in the formation of a two-helix bundle rather than the desired four-helix bundle. To correct this, we crafted a new construct, model 6c, which introduces an α-helix (PDB entry 2zta, residues 2–32) capable of dimerizing with its counterpart and replacing the original α-helix (PDB entry 2o6n) at the N-terminus of the DARPin [Fig. 1 ▸(*d*), Supplementary Fig. S2 and Sections S1 and S2]. To facilitate two-helix bundle formation, we incorporated a glycine–serine (GS) repeat linker that extends 12 Å beyond its predecessor. Additional enhancements included strategic mutations at the apoferritin–DARPin interface designed to bolster the stability of the DARPin component (see Sections S1 and S2).

**Figure 2:**
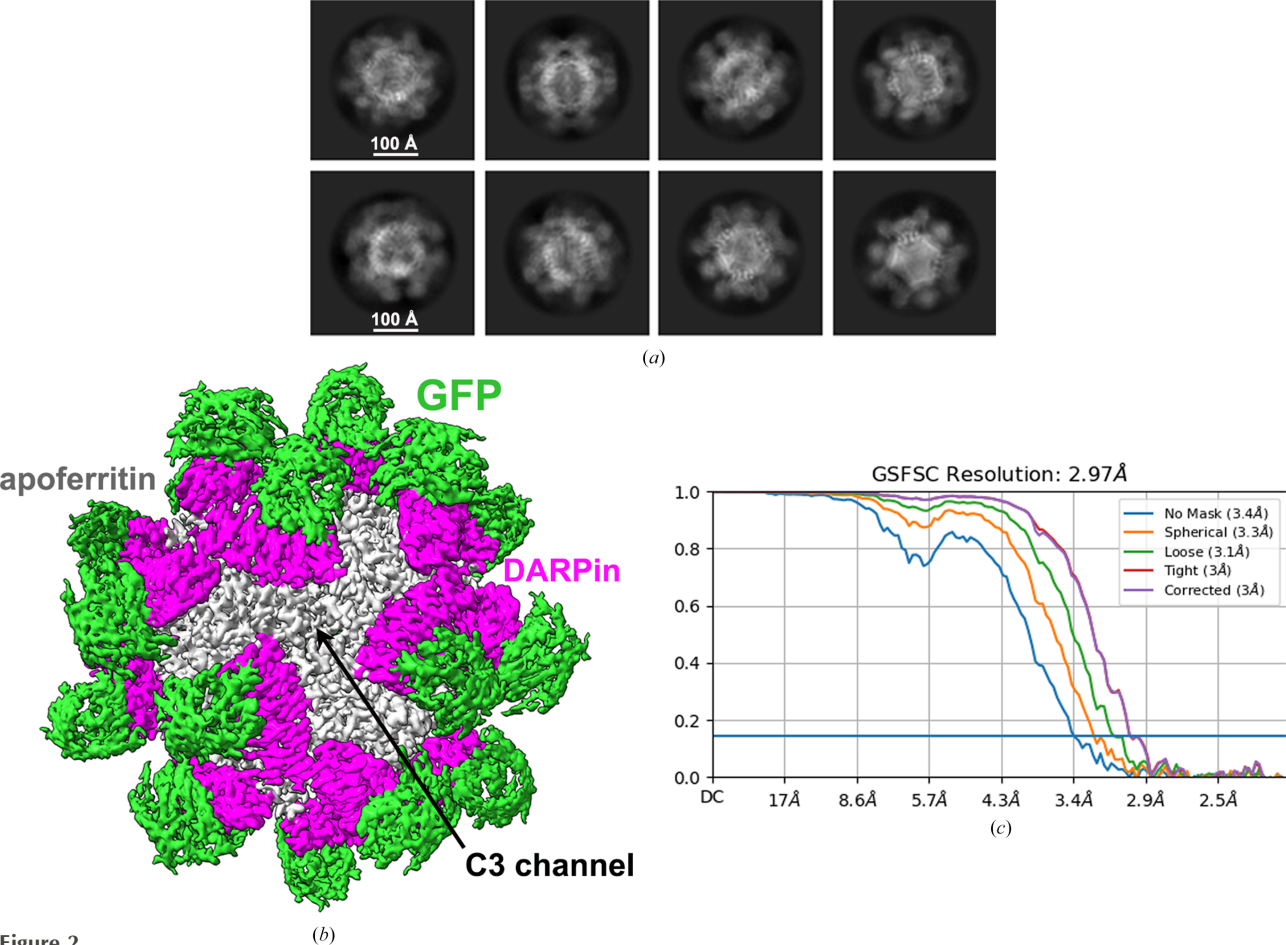
Cryo-EM data for the DARPin–apoferritin scaffold (model 6c) bound to GFP. (*a*) The 2D class averages display well aligned 24-mer cores along with clear density for the fused 17 kDa DARPin and the attached 27 kDa GFP. (*b*) A composite cryo-EM map, following 3D refinements, reveals the GFP attached to a rigid imaging scaffold. (*c*) The corresponding gold-standard Fourier shell correlation (FSC) curves are presented, achieving a resolution of 2.97 Å based on a correlation threshold of 0.143.

Negative-stain EM analysis of model 6c showed peripheral densities around apoferritin, a clear sign of the DARPin and its bound GFP [Supplementary Fig. S3(*a*)]. The single-particle cryo-EM raw images and subsequent 2D class averages depicted three distinct layers: the outer GFP shell, the middle adaptor protein DARPin and the inner apoferritin shell, perfectly in line with our design goals [Fig. 2 ▸(*a*)]. Single-particle analysis unveiled the 3D structure of the GFP–DARPin–apoferritin complex, achieving an impressive overall resolution of 2.97 Å [Figs. 2 ▸(*b*) and 2 ▸(*c*) and Supplementary Table S1]. The sharpened density map showcased the strong density of the apoferritin core, along with distinct density for the DARPin and its associated GFP molecules. Dimeric DARPins were observed encircling the C3 channel of apoferritin, while the GFP molecules firmly anchored to their respective DARPins on the outer surface of the scaffold [Fig. 2 ▸(*b*)].

**Figure 3:**
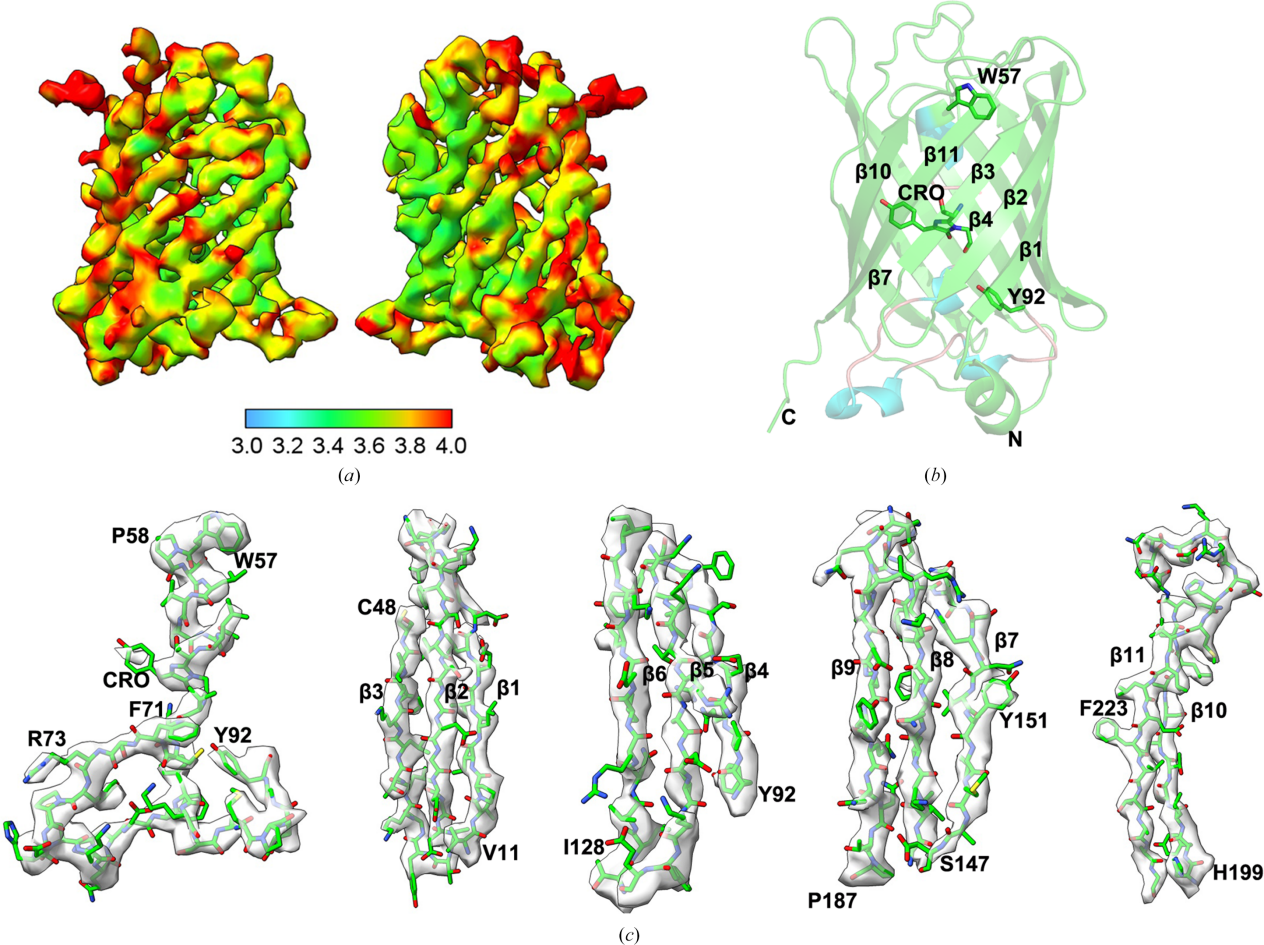
Near-atomic resolution map of the cargo protein GFP. (*a*) Two side views of the local resolution density map, showing bound GFP at a resolution of 3.47 Å, are presented, with each view rotated 180° relative to the other. This map was generated following multi-body refinement using *RELION* 3.1.2. (*b*) A ribbon diagram illustrates the GFP structure, where the helix (Trp57–Tyr92) along the central axis of the β-barrel (β1–β11) is interrupted by the chromophore (CRO) depicted as green sticks. (*c*) Focused views of the density map cover all GFP β-strands and the GFP chromophore, including its surrounding amino acids.

Post-processing and multibody refinement led to a final density map that portrayed a GFP molecule attached to a ‘dimeric’ DARPin with an overall resolution of 3.47 Å [Fig. 3 ▸(*a*), Supplementary Table S1]. This analysis underscored the key structural elements, especially the improved resolution on the GFP side interfacing with the DARPin. In higher resolution regions, the β-strands of GFP, the α-helices of the DARPin and the side chains of the residues were clearly defined in the sharpened density map (Supplementary Fig. S4). The atomic details of the β1–β3 and β7–β11 side chains, along with the backbone, are essentially clarified. For β4–β6, aside from a few amino-acid residues, the majority of the main and side chains are also clearly presented. The GFP chromophore and its adjacent amino-acid residues were detailed at near-atomic precision, setting a new benchmark for cryo-EM imaging of small proteins [Figs. 3 ▸(*b*) and 3 ▸(*c*)].

To evaluate the impact of scaffold binding on the structure of the protein, we compared the coordinates of the bound protein with those of unbound GFP (PDB entry 2b3q; Pédelacq *et al.*, 2006 ▸). The attachment of GFP to the DARPin induced no significant alterations to the backbone structure, with a root-mean-square (r.m.s.) deviation of just 0.646 Å. For comprehensive data-quality and model-refinement statistics, refer to Supplementary Table S1.

**Figure 4:**
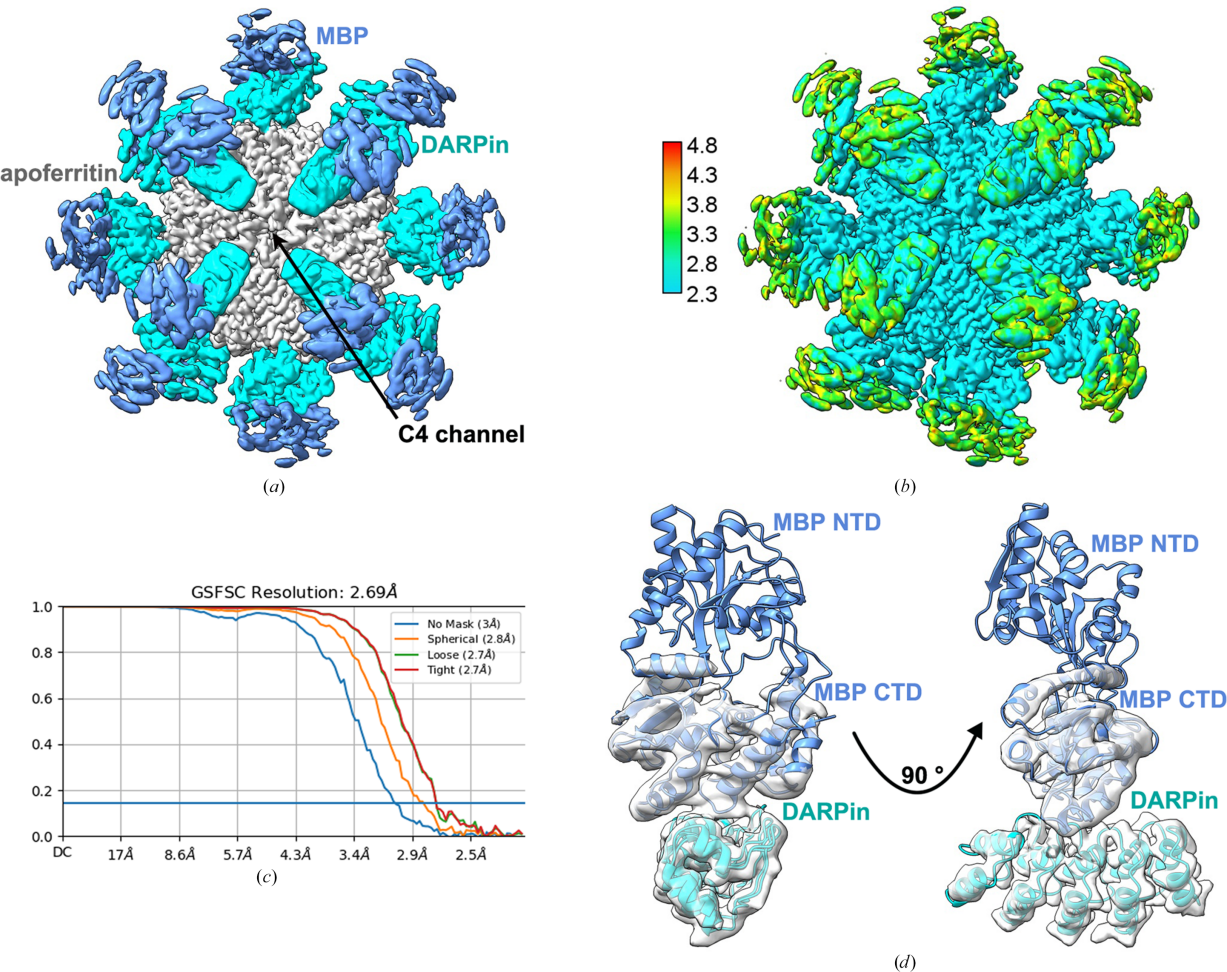
Cryo-EM data of the DARPin–apoferritin scaffold (model 4) complexed with MBP. (*a*) The consensus cryo-EM map, resulting from 3D refinements, reveals MBP attached to a stabilized imaging scaffold. (*b*) The local resolution map distinctly presents the apoferritin core at atomic resolutions close to 2.3 Å and the MBP C-terminal domain (CTD) with a near-atomic resolution of approximately 3.8 Å. Furthermore, the resolutions for MBP N-terminal domain (NTD) regions away from the DARPin exceed 4 Å. (*c*) The corresponding gold-standard Fourier shell correlation (FSC) curves are presented, achieving a resolution of 2.69 Å based on a correlation threshold of 0.143. (*d*) The density fits of DARPin and its bound MBP in two different views, which are related by a 90° rotation, are highlighted.

To demonstrate the versatility of our scaffold system, we aimed to retain the amino-acid sequence of the base protein, altering only the variable region of the DARPin in model 6c to target a distinct binding protein. We selected MBP (45 kDa) as a new target, given its larger size. However, the MBP-bound DARPin–apoferritin complex did not achieve the desired 24-mer structures, likely because of the strong interaction between the DARPin and apoferritin, as well as between neighboring DARPin molecules, similar to as observed in the GFP-model 6c complex. Comparative analysis revealed that model 4 exhibited significantly more 24-mer structures than model 6c. The ample space between the DARPin and apoferritin in model 4 allows the DARPin to undergo conformational changes upon binding to the target protein. This characteristic led us to choose model 4 with an anti-MBP DARPin for effective binding (see Section S2). Negative-stain EM micrographs revealed additional densities around the apoferritin, indicating the presence of the DARPin and its bound MBP [Supplementary Fig. S3(*b*)]. For 3D structural analysis, we collected 2347 cryo-EM images of the complex under cryogenic conditions using a Titan Krios microscope. After initial data processing, a significant number of particles were selected for 3D analysis based on their 2D class averages. The initial 3D reconstruction, performed using *cryoSPARC* 3.3.2 and targeting the symmetric core of the scaffold with octahedral (*O*) symmetry, yielded an overall resolution of 2.69 Å, revealing distinct amino-acid side-chain features [Fig. 4 ▸(*a*)]. The median local resolution for the DARPin was 3.5 Å, ranging from 2.8 to 4.3 Å, while the local resolution for MBP adjacent to the DARPin was 3.8 Å, with resolution exceeding 4 Å further from the DARPin [Figs. 4 ▸(*b*) and 4 ▸(*c*)]. The C-terminal domain (CTD) of MBP was well defined, with the side contacting the DARPin showing higher resolution than the outward-facing side. In high-resolution areas, the α-helices in the MBP CTD were distinct and easily traceable.

However, the N-terminal domain (NTD) of MBP was not visible in the sharpened density map [Fig. 4 ▸(*d*)]. This discrepancy is primarily attributed to the flexible hinge region connecting the NTD and CTD of MBP (Binz *et al.*, 2004 ▸). Additionally, the orientation and fewer contacts of the DARPin component with apoferritin in model 4 compared with those in model 6c may also contribute to the observed differences in resolution.

## 4. Discussion

In our quest to elucidate the structures of small proteins, we undertook a rigorous screening process, evaluating a diverse array of DARPin–apoferritin scaffold candidates for their non­covalent binding capabilities to these small cargo proteins, with the ultimate goal of facilitating structural analysis via single-particle cryo-EM. From this diverse pool, two constructs, model 4 and model 6c, emerged as particularly promising. Model 6c notably succeeded in determining the cryo-EM structure of our target protein, GFP, with such clarity that the side-chain density for nearly all amino-acid residues was discernible: a significant achievement in the realm of structural biology. Similarly, model 4 revealed the cargo protein MBP with a distinct and traceable C-terminal domain (CTD), further underscoring the potential of our scaffolds.

Recent studies have demonstrated the potential of engineered scaffolding systems, including *D*2-symmetric aldolase (Yao *et al.*, 2019 ▸), dihedral symmetric protein assemblies (Vulovic *et al.*, 2021 ▸) and ingeniously designed tetrahedral cages (Liu *et al.*, 2018 ▸, 2019 ▸; Castells-Graells *et al.*, 2023 ▸). All of these scaffolds have harnessed the power of DARPins as an adaptor to achieve near-atomic resolution imaging of small proteins such as GFP and K-Ras via cryo-EM. Our work not only provides detailed structural insights into GFP and MBP but also pioneers the integration of a highly symmetric octahedral (*O*) cage, apoferritin, into our scaffold design. The near-spherical shape of the scaffold offers a substantial advantage by mitigating the persistent issue of preferred particle orientation, a common challenge in cryo-EM, as demonstrated in the data processing in this study. The 24-subunit apoferritin framework allows the attachment of up to 24 target proteins without steric hindrance, an extraordinary feature. The presence of multiple target protein copies on each particle keeps these proteins at a safe distance from the air–water interface, a region known to induce unpredictable particle distribution, protein denaturation, complex dissociation and preferential orientation (Liu & Wang, 2023 ▸).

Moreover, our DARPin–apoferritin scaffold surpasses alternative approaches with its straightforward expression and purification processes, as well as the ease of DARPin replacement for targeting other proteins of interest, as demonstrated with GFP and MBP. A new binding DARPin could readily substitute that in model 4 or model 6c through sequence alignment, while retaining the mutations introduced in this study. Our analysis of the MBP–model 4 structure shows that the model 4 scaffold can accommodate targets significantly larger than MBP, with no steric hindrance between neighboring cargo-protein units, as shown by the clear separation within the model 4 particle (Fig. 4 ▸).

Furthermore, apoferritin, the base protein in our scaffold, has set a benchmark in cryo-EM by achieving an atomic resolution of 1.55 Å using only 22 000 particles, demonstrating its exceptional quality (Yip *et al.*, 2020 ▸). Our scaffold, with its high-resolution GFP structure, outperformed other reported scaffolds (Castells-Graells *et al.*, 2023 ▸; Liu *et al.*, 2019 ▸) by requiring the fewest particles, thus reducing the demands of image collection and data procession, and setting a new precedent in efficiency.

Moreover, our GFP–model 6c and MBP–model 4 molecules, with their larger particle sizes ranging from 20 to 30 nm, are ideally suitable for cryo-EM analysis. Considering the average ice thickness at the center of gold and carbon nanowire grids, which varies from 30 to 56 nm (Noble *et al.*, 2018 ▸), our protein-scaffold particles are efficiently embedded as a monolayer in vitreous ice when prepared using standard cryo-EM grid-preparation techniques. This method substantially minimizes variations in defocus levels and eliminates the possibility of projection overlaps.

Taking these factors into account, our DARPin–apoferritin scaffold emerges as an optimal solution for resolving the structures of small proteins via cryo-EM. The successful application of this scaffold to the cryo-EM analysis of MBP (45 kDa) paves the way for employing this methodology on protein targets that pose a challenge for NMR or crystallo­graphic analysis, particularly for proteins larger than 50 kDa that struggle to form well diffracting crystals. Even a low-resolution cryo-EM structure can provide invaluable functional insights or act as a springboard for subsequent crystallographic or NMR studies.

Like other scaffolds, our system has limitations. Both the GFP– and MBP–DARPin–apoferrin complexes exhibit lower resolution of the outer-shell target protein compared with the inner-shell apoferritin. This phenomenon has also been observed in other symmetric scaffolds (Yao *et al.*, 2019 ▸; Vulovic *et al.*, 2021 ▸; Castells-Graells *et al.*, 2023 ▸). In addition to the rigidity of the scaffold pursued in this study, the binding affinity between the DARPin and the target protein, as well as the inherent rigidity of the target protein itself, may also influence the resolution of the complex. Surface plasmon resonance (SPR) results indicate that the binding affinity of GFP to DARPin (PDB entry 5ma8) is 303 p*M* (Hansen *et al.*, 2017 ▸), whereas that of MBP to DARPin (PDB entry 1svx) is 4.4 n*M* (Binz *et al.*, 2004 ▸). This difference may partially explain why GFP achieves higher resolution than MBP in the scaffold. A flexible hinge region links the N- and C-terminal domains of MBP, with the DARPin-contacting C-terminal domain exhibiting higher resolution. This underscores the importance of the rigidity of the target protein in resolution improvement.

For future applications of this scaffold, DARPin screening is crucial for high-resolution structure determination of target proteins. Both the binding affinity and the binding epitope are important factors. DARPins that bind to the midpoint rather than the terminus of the target protein with high affinity are more likely to yield high-resolution structures. Additionally, protein engineering to enhance the rigidity of the target protein, such as loop mutations, deletions or truncations, is essential. Moving forward, structure determination of target proteins with unknown structures and membrane proteins is vital for showcasing the versatility and imaging advantages of cryo-EM scaffolding systems such as those we have discussed. The continuous refinement of these systems is pivotal for realizing the ambitious goals of high-throughput structure determination.

## Supplementary Material

PDB reference: DARPin–GFP, 9irv

PDB reference: MBP–DARPin–apoferritin, 9ivp

PDB reference: GFP–DARPin–apoferritin, 9j48

EMDB reference: DARPin–GFP, EMD-60822

EMDB reference: MBP–DARPin–apoferritin, EMD-60931

EMDB reference: GFP–DARPin–apoferritin, EMD-61130

Supplementary figures and tables, and design procedures for DARPin-apoferritin scaffolds. DOI: 10.1107/S2052252525003021/eh5021sup1.pdf
