# Supplementary material for: A large, general and modular DARPin–apoferritin scaffold enables the visualization of small proteins by cryo-EM
Source: IUCrJ. 2025 Apr 25;12(Pt 3):393–402. doi: 10.1107/S2052252525003021 (PMC12044855; doi:10.1107/S2052252525003021)
Supplement: Supplementary file 1 [file m-12-00393-sup1.pdf]

IUCrJ

Volume 12 (2025)

Supporting information for article:

A large, general and modular DARPin–apoferritin scaffold enables the visualization of small proteins by cryo-EM

Xin Lu, Ming Yan, Yang Cai, Xi Song, Huan Chen, Mengtan Du, Zhenyi Wang, Jia'an Li, Liwen Niu, Fuxing Zeng, Quan Hao and Hongmin Zhang

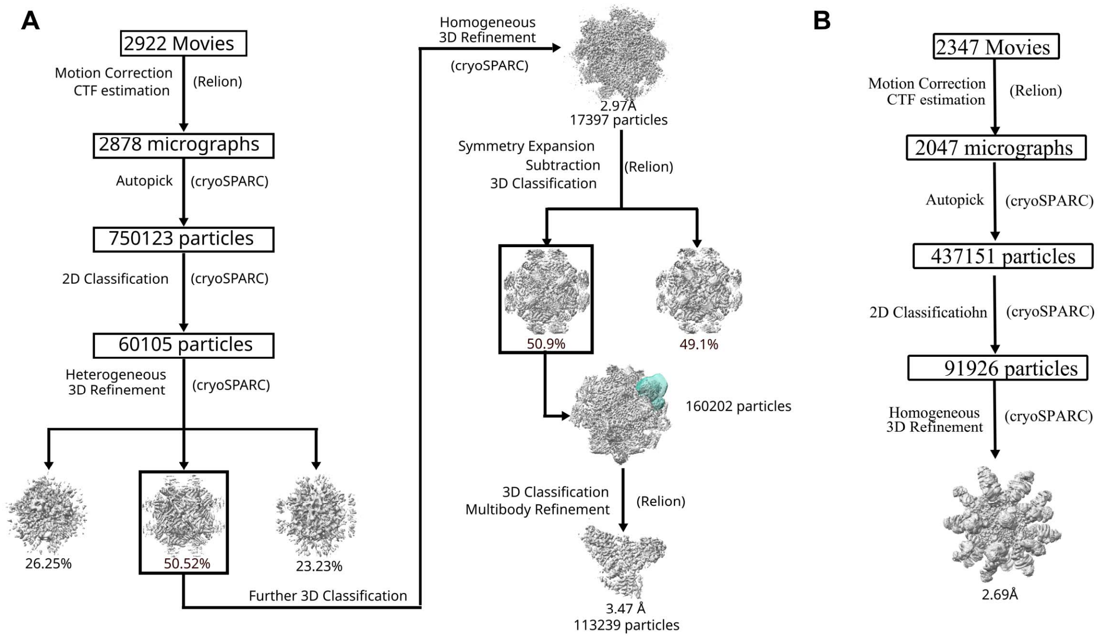

Figure S1 Cryo-EM data collection and image processing procedure for high-resolution reconstruction of GFP:Model 6c (A) and MBP-Model 4 (B) complexes. (see Methods).

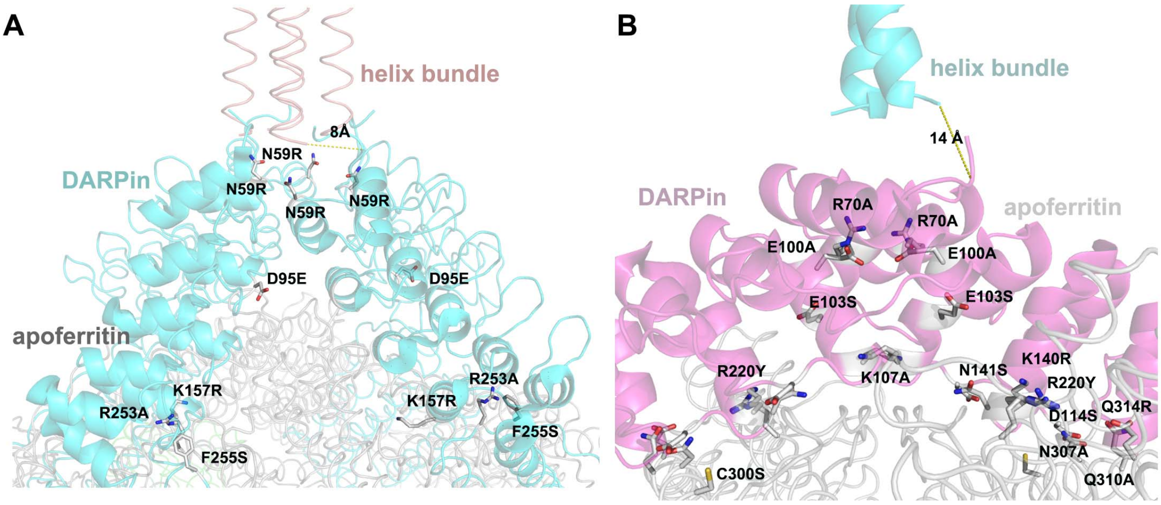

Figure S2 Docking model of helix bundle-DARPin-apoferritin-helix bundle scaffold. (A) A detailed view of docking Model 4 and (B) Model 6c, which were specifically designed and characterized in this study. The helix bundle portion was designed to stabilize the scaffold, as illustrated in the figure. Additionally, several mutations were introduced at the interface between DARPin and apoferritin to enhance rigidity and minimize potential steric clashes.

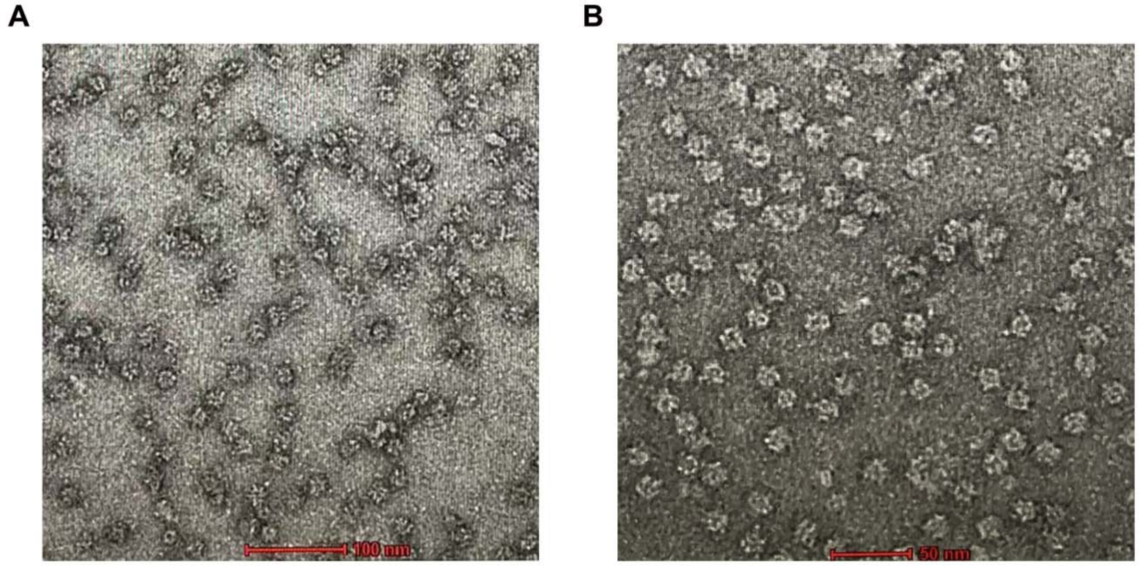

Figure S3 The negative stain EM micrographs of GFP-model 6c(A) and MBP-model 4 (B).

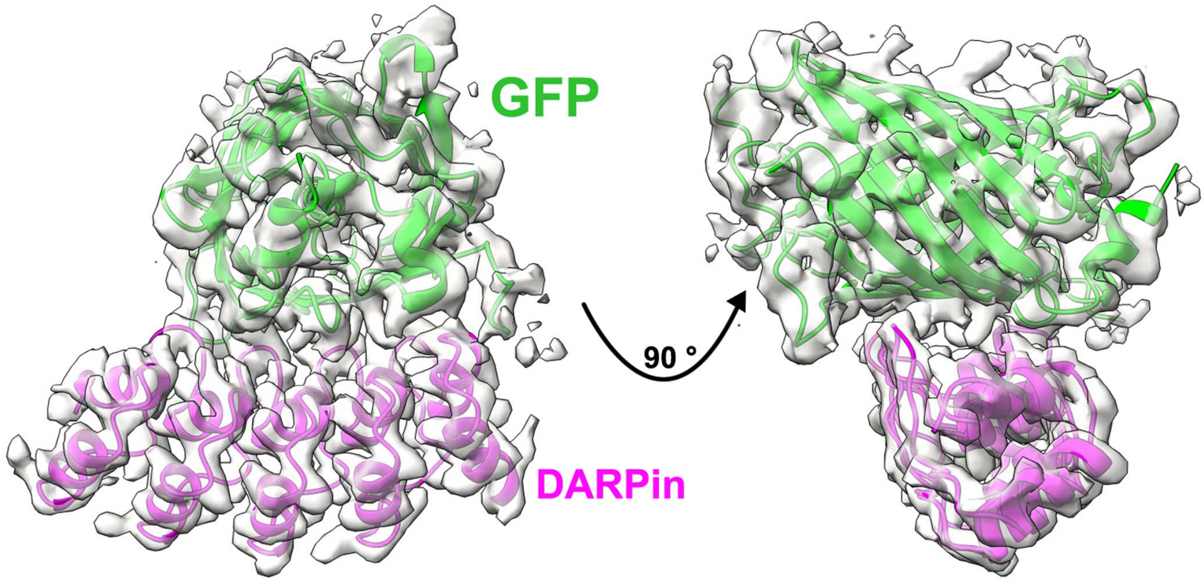

Figure S4 Overview of the final density map for DARPin-GFP. The final density map provides a comprehensive view of the DARPin and its bound GFP protein, depicted from two distinct perspectives separated by a 90° rotation.

Table S1 Cryo-EM data collection, processing and modeling

Data Collection			
Microscope	FEI TITAN KRIOS		
Imaging Mode	Microprobe (EF)		
Detector	Gatan K2 Summit		
Voltage (kV)	300		
Magnification	130,000 x		
Pixel Size (Å /pixel)	1.076		
Total electron dose (e-/Å2)	50		
Frame Rate (frames/second)	6.3		
Defocus range (µm)	-0.8 ~ -2.0		
Processing			
Map Description	Overall structure of GFP-DARPin-apoferritin	Overall structure of MBP-DARPin-apoferritin	Local-refined MAP of DARPin-GFP
EMDB Reference Number	EMD-61130	EMD-60931	EMD-60822
Softwares	cryoSPARC-3.3.2	cryoSPARC-3.3.2	cryoSPARC-3.3.2, Relion-3.1.2
Total Images	2922	2347	
Total Extracted Particles	750,123	437,151	750,123
Particles for Symmetry Expansion			17,397
Particles for Multibody Refinement			113,239*
Particles for Final Reconstruction	17,397	91,926	113,239*
Refinement Approach	3D Refinement & Reconstruction	3D Refinement & Reconstruction	Multi-body Refinement
Map Symmetry	O	O	C1

Map Resolution(Å)	2.97	2.69	3.47
FSC Threshold	0.143	0.143	0.143
Modeling			
PDB Reference Number	9J48	9IVP	9IRV
Softwares	Coot-0.9, Phenix-1.16	Coot-0.9, Phenix-1.16	Coot-0.9, Phenix-1.16
Initial Models	PDB-3AJO, 5MA8, 6NHV	PDB-3AJO, 1SVX,5M13	PDB-5MA8, 6NHV
Model Resolution	3.0	3.0	3.5
Validation			
MolProbity score	1.88	1.63	1.55
All-atom clashscore	10.34	7.44	6.28
Rotamers outliers (%)	0.00	0.36	0.00
CBeta Outliers(%)	0.00	0.00	0.00
CC(Mask)	0.79	0.57	0.80
R.M.S deviations			
Bonds lengths (Å)	0.009	0.007	0.009
Bonds angles (°)	1.123	1.099	1.229
Ramachandran plot statistics			
Preferred (%)	95.03	96.56	96.72
Allowed (%)	4.97	3.44	3.28
Outlier (%)	0.00	0.00	0.00

* The particle stack originates from an expansion of original particles with O symmetry.

Table S2 Characterization of six DARPin-apoferritin scaffolds (models 1-6).

Model	Expression level [a]	Polymerization	Negative staining EM [b]	DARPin resolution (Å) [c]	GFP resolution (Å) [d]
1	High	24-mer	Few	-	-
2	High	24-mer	Few	-	-
3	High	24-mer	Few	>5	-
4	High	24-mer	Regular	<4	3.5-4
5	Low	-	-	-	-
6	High	24-mer	Regular	>4	-

[a] Protein expression levels were meticulously quantified using SDS-PAGE and NanoDrop equipment. A designation of "high" signifies that the protein is expressed in sufficient quantities and with high quality, making it suitable for subsequent experimental procedures.

[b] "Regular" characterizes the majority of particles that exhibit ring structures with a diameter of 120 Å, marked by an increased density at the periphery of the shell. Conversely, a "few" classifications indicates that the majority of particles exhibit irregular structures, deviating from the expected ring formation.

[c, d] The resolution values were ascertained based on the local resolution estimates provided by cryoSPARC- 3.3.2.

S1. Design Procedure of Model 4 and Model 6c

To develop an effective DARPin-apoferritin scaffold, we initiated by fusing α-helices at the termini of anti-GFP DARPin (PDB ID: 5MA8) and apoferritin (PDB ID: 3AJO). During this process, the N-terminal loop of apoferritin was truncated to facilitate a continuous connection to the C-terminus of DARPin, with the C-terminal loop of DARPin also deleted. Cryo-EM analysis revealed that while the apoferritin portion of the 5MA8-3AJO scaffold exhibited high-resolution features, the DARPin portion was nearly invisible in the sharpened density map (data not shown). To address this challenge, we designed a docking model. A long α-

helix (PDB ID: 2O6N, residues 2–34) was added to the N-terminus of DARPin to form a potential four-helix bundle motif with three other α-helices in the scaffold, aiming to enhance the rigidity of the DARPin portion. A glycine-serine (GS) repeat linker (GGGGGS) was employed to connect this long α-helix to the N-terminus of DARPin based on their spatial distance. Additionally, N59R, D95E, and K157R mutations were introduced in the DARPin portion to enhance DARPin-DARPin and DARPin-apoferritin interactions. In the apoferritin portion, mutations R253A and F255S were implemented to minimize steric clashes between DARPin and apoferritin (Figure S2A). This constituted the initial design procedure for Model 4. However, static light scattering analysis indicated that the potential four-helix bundle motifs disrupted the 24-mer formation of the scaffold (data not shown). After iterative optimization, we replaced the long α-helix (30 aa) previously at the N-terminus of DARPin with an α-helical element at the N-terminus of DARPin (PDB ID: 2O6N, residues 18–34) and the C-terminus of apoferritin (PDB ID: 2O6N, residues 2–17). This modification enabled the scaffold to form a stable 24-mer (Figure 1C).

For Model 6, based on our docking model, (Figure S2B), an α-helix (PDB ID: 2ZTA, residues 2–34) was added to the N-terminus of DARPin to form a potential two-helix bundle motif with another α-helix in the scaffold, with the goal of enhancing the rigidity of the DARPin portion. A glycine-serine (GS) repeat linker (GGSGGSGG) was used to connect this α-helix to the N-terminus of DARPin, considering their spatial distance. To minimize steric clashes, most interface residues between DARPin-DARPin or DARPin-apoferritin were mutated to alanine or serine. Additionally, mutations R220Y/K140R were introduced to strengthen interactions at the DARPin-apoferritin interface.

S2. The chimeric design of three DARPin-apoferritin scaffolds specifically model 4 (for MBP or GFP binding) and model 6c (for GFP binding).

In these models, the DARPin segments are distinguished by cyan and magenta colors for model 4 and model 6c, respectively, while the apoferritin component is depicted in gray. Notably, the N-terminal segment of the DARPin exhibits a potential helix-bundle motif, highlighted in deep salmon: model 4 (PDB ID 2O6N, residues 18-34) and model 6c (PDB ID

2ZTA, residues 2-32), which parallels the motif found in the C-terminus of apoferritin (PDB ID 2O6N, residues 2-17), depicted in yellow. A glycine or glycine-serine repeat linker between DARPin and the potential N-terminal helix-bundle region, designed to enhance the formation of the helix-bundle motif, is highlighted in orange. Additionally, mutation sites engineered to stabilize the interface between apoferritin and DARPin are marked in red. Model 4 for MBP binding shows only few amino acid mutations (colored in purple) on the interface of DARPin interacting with MBP compared to model 4 for GFP binding. The linker to connect apoferritin and DARPin is colored in blue.

>Model 4 for GFP binding

MGHHHHHHGPGSAKEEILEEIKKAKQEIAGGGGGSELGKELLEAARAGQDDEVRILMARGAEVNAADDV
GVTPLHLAAQRGHLEIVEVLLKYGAEVNAADLWGQTPLHLAATAGHLEIVEVLLKNGADVNARDNIGHT
PLHLAAWAGHLEIVEVLLRYGADVEAQDKFGKTPFDLAIDNGNEDIAEVLQALLAINRQINLELYASYV
YLSMSYYFDRDDVALKNFAKYFLHQSHEEREHAEKLMKLQNQRGGAISLQDIKKPDCDDWESGLNAMEC
ALHLEKNVNQSLLELHKLATDCNDPHLCDFIETHYLNEQVKAIKELGDHVTNLRKMGAPESGLAEYLFD
KHTLGSGSGAEIEQAKKEIAYLIKK

>Model 4 for MBP binding

MGHHHHHHGPGSAKEEILEEIKKAKQEIAGGGGGSELGKELLEAARAGQDDEVRILMARGAEVNAADNT
GTTPLHLAAYSGHLEIVEVLLKYGAEVNAADVFGYTPLHLAAYWGHLEIVEVLLKNGADVNARDSDGMT
PLHLAAKWGHLEIVEVLLRYGADVEAQDKFGKTPFDLAIDNGNEDIAEVLQALLAINRQINLELYASYV
YLSMSYYFDRDDVALKNFAKYFLHQSHEEREHAEKLMKLQNQRGGAISLQDIKKPDCDDWESGLNAMEC
ALHLEKNVNQSLLELHKLATDCNDPHLCDFIETHYLNEQVKAIKELGDHVTNLRKMGAPESGLAEYLFD
KHTLGSGSGAEIEQAKKEIAYLIKK

>Model 6c
